# Genomic Analysis Reveals Subdivision of Black Rats (*Rattus rattus*) in India, Origin of the Worldwide Species Spread

**DOI:** 10.3390/genes13020267

**Published:** 2022-01-29

**Authors:** Mumtaz Baig, Sameera Farah, Ashwin Atkulwar, Jeremy B. Searle

**Affiliations:** 1 Laboratory of Molecular and Conservation Genetics, Department of Zoology, Govt. Vidarbha Institute of Science and Humanities, Amravati 444604, India; mumtaz@lmcg.in (M.B.); farahs@uoguelph.ca (S.F.); ashwin@lmcg.in (A.A.); 2 Department of Integrative Biology, University of Guelph, 50 Stone Road East, Guelph, ON N1G 2W1, Canada; 3 Department of Ecology and Evolutionary Biology, Cornell University, Corson Hall, Ithaca, NY 14853-2701, USA; 4 Department of Zoology, Amolakchand Mahavidyalaya, Godhani Road, Yavatmal 445001, India

**Keywords:** commensalism, Indian subcontinent, Indus civilization, invasive, population genomics

## Abstract

In contrast to the detailed and globally extensive studies on the spread of the commensal black rat, *Rattus rattus*, there has been relatively little work on the phylogeography of the species within India, from where this spread originated. Taking a genomic approach, we typed 27 *R. rattus* samples from Peninsular India using the genotyping-by-sequencing (GBS) method. Filtering and alignment of the FASTQ files yielded 1499 genome-wide SNPs. Phylogenomic tree reconstruction revealed a distinct subdivision in the *R. rattus* population, manifested as two clusters corresponding to the east and west coasts of India. We also identified signals of admixture between these two subpopulations, separated by an *Fst* of 0.20. This striking genomic difference between the east and west coast populations mirrors what has previously been described with mitochondrial DNA sequencing. It is notable that the west coast population of *R. rattus* has been spread globally, reflecting the origins of commensalism of the species in Western India and the subsequent transport by humans worldwide.

## 1. Introduction

The demographic history and origin of commensalism in rodents inevitably reflects the history of human settlements, sedentism, and ancient trades. Commensal rodents have been subject to detailed zooarchaeological studies, given this association with human history, and the profound influence of commensal rodents on human economics and health. Among rodents, black rats (*R. rattus*) and house mice (*Mus musculus*) are the world’s most widespread and destructive invasive species [1,2]. Besides causing damage to crop and agricultural products every year, *R. rattus* serves as the carrier of zoonotic diseases, such as plague, typhoid and leptospirosis [3]. Based on mitochondrial DNA (mtDNA) analyses, phylogeographic studies on *R. rattus* have revealed a long history of commensalism and maritime transport by humans [4,5]. Both archaeological and genetic evidence point to India as the origin of *R. rattus* and its dispersal outside of the subcontinent by maritime activities operating from its coasts [4,6,7,8]. Considering that the commensalism of *R. rattus* originated in India [8], geography and human history are key. The Indian Peninsula borders the Indian Ocean along two coastlines, the west and east coasts, and there is the Gangetic Plain that separates the Indian Peninsula from the Himalayas, which has been considered the most fertile region in mainland India since historic times. In the northwest of the Indian subcontinent, the rise of the ~5 kya Indus civilization is considered a major centre that provided an impetus for the origin of the earliest human civilization in South Asia [9,10], and the available evidence suggests that *R. rattus* first became commensal with the rise of that civilization [4,6,8]. In this way, the beginnings of agriculture, with subsequent transformation to village and city life, most probably paved the way to commensalism for the *R. rattus* population living in the northwest of the subcontinent. Clearly, India is very important in understanding commensalism in *R. rattus*, with a need for further studies.

**Figure 1:**
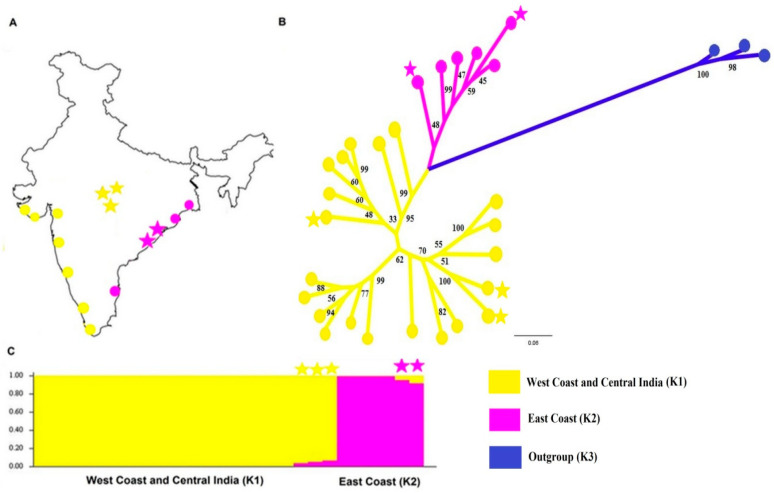
(**A**) Sampling locations of *R. rattus* across India (yellow circles depict west coast and central India locations, while pink circles indicate east coast locations; stars indicate locations highlighted in (**C**)). (**B**) SNP-based maximum likelihood phylogenomic tree constructed using 27 *R. rattus* samples. Clade K1 includes samples from the west coast and central India while clade K2 includes samples from the east coast. Similarly, clade K3 comprises 3 *M. m.* *castaneus* samples used as the outgroup. (**C**) STRUCTURE plot illustrating the population structure in *R. rattus*, sampled from the west coast, east coast and central India.

Genetics has a long history of applications in zooarcheological studies, including providing insights into ancient human migrations and colonizations, and the domestication of animals. Previous research on *M. musculus* exemplifies how commensal rodents can be used as a model biomarker to study ancient human history. Several mtDNA-based studies elucidate the maritime transportation of *M. musculus* by Vikings [11,12,13]. Genetic evidence in support of the transport of house mice to Australia from the British Isles in the late 18th and 19th centuries by maritime activities has also been obtained [14]. The present study examines the phylogeography and population genomics of *R. rattus* in India using genome-wide SNP data. So far, most of the phylogeographic studies on *R. rattus* have focused on mtDNA, and four mitochondrial lineages (I–IV) of the *R. rattus* complex (RrC) have been reported in mainland Asia [4,15], with commensalism associated with the RrC lineage-I. Despite large sample sizes, most of the studies attempted on *R. rattus* outside India demonstrate low diversity [16,17,18]. Taking into consideration the power of genomics and the role of India in the dispersal of *R. rattus* in Eurasia and elsewhere, understanding the genetic diversity of *R. rattus* within Peninsular India is of the utmost importance. Taking a genome-wide approach, we genotyped *R. rattus* samples from a wide range of localities in India, with emphasis on the west and east coasts, which are regions that allowed the spread of commensal *R. rattus* from Peninsular India (Figure 1A). Since prehistoric times, both the west and east coasts witnessed flourishing of human settlements and kingdoms that had maritime links with West and Southeast Asia [19]. The uses of population genomic approaches have started to provide better insights into the complex histories of invasions of species worldwide [20,21,22]. Thus, the main objective of this study was to use the combined power of genome-wide SNPs to decipher the population structure in spatially distributed *R. rattus* populations across India, and to provide insight into commensalism in the *R. rattus* population in light of the knowledge of the archaeology and history of India.

## 2. Materials and Methods

### 2.1. Sample Collection and DNA Extraction

Thirty-four *R. rattus* were live trapped from different locations in India (Figure 1A and Table S1). After removing the tail tip, all sampled *R. rattus* individuals were released. The same individuals were sequenced earlier by us for Cyt b and D-loop sequencing [8]. The study was approved by the Animal Ethical Committee of the Govt. Vidarbha Institute of Science and Humanities, Amravati, India. All tail tips were preserved in ethanol until subject to genomic DNA extraction using Qiagen Blood and Tissue DNA extraction kit (Qiagen, Germantown, MD, USA). Sampling of rats was carried out in rural communities so as to avoid the possibility of admixture by recent arrival in the cities through transport by trains and buses. Three house mice, *M. m. castaneus*, were also sampled to be used as an outgroup.

### 2.2. DNA Sequencing and SNP Genotyping

A genotyping-by-sequencing (GBS) approach was used to prepare the libraries and genotype genome-wide SNPs for the 34 *R. rattus* and 3 *M. musculus* samples. GBS was performed at the Cornell Genomic Diversity Facility following the protocol described by [21,22,23]. After removing adaptors and filtering the FASTQ file, the *R. rattus* sequences were aligned with the reference genome for SNP calling in TASSEL-GBS pipeline version 5 [24]. A minimum base call of five reads and a maximum locus missing data of 20% were defined. We used the whole genome of *Rattus norvegicus* (GCA_000317375) as a reference genome for SNP calling. After SNP calling, the metadata were exported in a VCF file format for downstream analyses using TASSEL 5 Standalone version [24]. Seven samples were omitted due to poor typing quality. Thus, a resulting vcf file comprising 27 *R. rattus* and 3 *M. m. castaneus* samples was processed for further analysis.

### 2.3. Phylogenomic Tree Construction

Initially, we used a distance matrix to build a simple neighbour-joining phylogenetic tree within the TASSEL program. To further revisit the phylogenetic relationship, we constructed a maximum likelihood (ML) tree using RAxML v8.2.X [25] and further modified the tree in Figtree 1.4.2 [26].

### 2.4. Genetic Subdivision

The genetic subdivision of *R. rattus* in India was explored by Bayesian clustering using the program STRUCTURE 2.3.2 [27,28]. The number of clusters (K) was identified by running the program for 1 million MCMC chains, following a burn-in of 1 million clusters. A batch run approach was used with 5 iterations for each cluster ranging from K = 1 to 4 incorporating admixture with the allele frequency-correlated model. The most probable number of clusters was selected using the second-order rate of change of log probability of the data between successive values of K (∆K) [29] in the program STRUCTURE HARVESTER v0.6.8 [30]. The whole analysis was repeated three times to confirm the accuracy of the analysis. ARLEQUIN 3.5.1 [31] was used to further examine genetic differentiation using *F_ST_*.

## 3. Results

### 3.1. Genomic Variation and Diversity Indices

The *R. rattus* samples, originating from 16 locations from the west coast, east coast, and central India, were genotyped to yield 1499 genome-wide SNPs. The average minor allele frequencies were calculated as 0.1750, and the heterozygous proportion was found to be 0.2944.

### 3.2. Phylogenomics and Genetic Subdivision in R. rattus

With strong bootstrap support, the genome-wide SNPs resulted in an ML tree, which showed the splitting of *R. rattus* samples into two distinct clusters, corresponding to the east and west coasts of India (Figure 1A,B). Moreover, the *R. rattus* sampled from central India were grouped with west coast samples. In addition to this, the analysis in STRUCTURE 2.3.2 reconfirms the clustering pattern revealed by the phylogenomic tree (Figure 1C). The highest value of ∆K for K *=* 2, detected by Evanno’s method, reveals the partitioning of the contemporary *R. rattus* population in India into two lineages (Figure S1 and Table S2). Additionally, the average genetic differentiation (*F_st_*) calculated for these two clusters was 0.204, indicating strong disparity between the spatially separated *R. rattus* populations. Of note, genetic signals of admixture were also detected in five *R. rattus* samples obtained from central India and the east coast (Figure 1C).

## 4. Discussion

In recent years, rodents, including house mice, *M. musculus*, and black rats, *R. rattus*, have been studied extensively as a proxy to understand ancient human settlements and movements. Considering the lack of information and importance of understanding the genomic diversity of *R. rattus* within India, we used the genotyping-by-sequencing (GBS) approach to study samples obtained from the west and east coasts and central India to understand their genetic relationship. Both our phylogenomic tree and clustering analysis show distinct subdivisions in the *R. rattus* population, corresponding to the west and east coasts. This genomic differentiation in the *R. rattus* population within Peninsular India corroborates the substantial mitochondrial diversity and differentiation that we have previously reported in the *R. rattus* population in India [8]. Both in our previous mitochondrial DNA study and in the current genomic analysis, we found clear divergence between the west coast and the east coast. Although we have not been able to perform accurate dating for either of our analyses, the close correspondence between the genomic and mitochondrial data suggests substantial differentiation between the west coast and east coast populations that would have occurred at a time well prior to the human settlements and the origin of commensalism. This is substantiated by the mitochondrial DNA tree (Figure 2 in reference [8]), which shows a level of differentiation between the west coast and east coast populations of a similar magnitude as between major phylogenetic lineages within *R. rattus* previously described in [4]. In Peninsular India, the west and east regions fall under two different ecological zones [32] that might have shaped the genome of *R. rattus* differently.

Considering the first association between *R. rattus* and humans, the advent of agriculture followed by a village/urban way of life in the northwest of the Indian subcontinent is the most plausible explanation for the origin of commensalism in the wild-living *R. rattus* population, and, once commensal, humans unintentionally moved them around [10,33]. Mehrgarh, a 7 kya site, was the epicentre of the origin of Indus valley civilization in the northwest of the subcontinent [9,34]. Most probably, it was in this region where commensalism first originated in *R. rattus* and further spread to other parts of the sub-continent. Many sites, ranging from early (~7–6.5 kya) to late (~5–2.8 kya) Indus, have been excavated in India [35]. Importantly, ten of the samples genotyped in this study originated from the northwest (Table S1: Rr1, Rr2, Rr3, Rr4, Rr5, Rr6, Rr9, Rr12, Rr13, Rr14). At many of the Neolithic Indus valley sites in the northwest, commensal *R. rattus* bones have been found and recorded [36,37]. In terms of early human settlements, compared to the northwest, the eastern and central Indian regions are less explored. However, a few studies suggest the origin of agriculture in other regions of India, including the Deccan Plateau in central India, as well as in the east (i.e., parts of present-day Bihar and Orissa), to 4.8–3.5 kya [32,38]. In addition, the presence of Neolithic practices of Austro-Asiatic linguistic tribes, such as Munda, Korku, and Santhal, in central and Eastern India is well documented [39].

Of the two lineages of *R. rattus* found on the east and west coasts, the basal positions of east coast *R. rattus* populations in both mtDNA [8] and genomic phylogenies indicate them to be ancestral. For mtDNA, there is evidence of greater genetic diversity in the east coast population, and the west coast population may be derived from the east coast population by having spread westwards well before *R. rattus* became commensal [8]. The signatures of the genetic admixture found in the *R. rattus* samples of central India and the east coast could be the outcome of ancient hybridization between the lineages, or it might have resulted from the intermixing of two lineages in this region, as a result of human transport. Considering the intermediate position of central India between the east and west coasts, admixture in this region is to be expected, although the *R. rattus* from central India predominantly have a west coast genetic signature. The contribution of the east coast rat genome to the genome in these central India individuals is very small. Given this characteristic of the central India *R. rattus*, and the relatively close proximity of the central India and some east coast populations, it is not surprising that there is admixture in both.

The genomic studies reported here confirm the coherence of the *R. rattus* population on the west coast of India already suggested by mtDNA analysis [8]. It is here that *R. rattus* became commensal with humans, and from here that *R. rattus* spread globally [4,8]. The mtDNA diversity for globally distributed *R. rattus* is low, as would be expected when largely derived from this one source area (the west coast of India) [4,8]. Interestingly, that is also true within that source area, also consistent with the human-mediated spread of commensal *R. rattus* throughout the west coast from a site of initial commensalism in the Indus valley. This could be the reason for the low mtDNA diversity on the west coast, rather than ancient derivation from the east coast populations. The present study indicates that the globally distributed *R. rattus* is not only derived from one particular geographically localised mtDNA lineage [8], it is derived from a population on the west coast of India that has distinct nuclear genome characteristics. This is evident because we have sampled all the way along the west coast of India, and these rats show genomic coherence, but are consistently different from those along the east coast (Figure 1). It is striking that the *R. rattus* on the east coast of India are also commensal, but, as we have said, they are genomically different from those on the west coast, and more genetically diverse in mtDNA (see Figure 5 in reference [8]). Similarly, there is not the same indication of a rapid spread from a single commensal source area. More sampling and genomic analyses are needed to fully understand this difference between the east and west coast. It would also be informative to include studies of ancient DNA from Indus and other Neolithic sites in the subcontinent to incorporate a time dimension into the work. India, as the origin of the spread of *R. rattus*, has much to offer to our understanding of the phylogeography of this species. It should be noted that this phylogeography and, in particular, knowledge of the source area is key information when trying to elucidate the invasiveness and other properties of the introduced populations of black rats, e.g., as vectors of human disease [40]. Taking this more broadly, it is important to emphasize that genetic and genomic studies of the black rat can help our understanding of various aspects of this most destructive, invasive mammal, and its management, as illustrated by recent studies defining eradication units [41,42,43].

## 5. Conclusions

India is crucial as the source area of the global spread of the black rat, *R. rattus*. Here we conduct the first population genomic study of Indian *R. rattus*, and demonstrate a similar genetic subdivision into east and west coast populations as found with mt DNA. Thus, the *R. rattus* worldwide range expansion originated from a west coast Indian population with defined nuclear genome characteristics as well as its well-known mt DNA characteristics. This understanding is valuable not only for future genome-based phylogeographic studies but also with respect genetic approaches to managing *R. rattus* as a devastating pest worldwide.

## Data Availability

The VCF file is provided as Electronic Supplementary Material.

## Supplementary Materials

The following supporting information can be downloaded at: https://www.mdpi.com/article/10.3390/genes13020267/s1: Figure S1: a STRUCTURE HARVESTER output illustrating the values of delta K for the most probable number of clusters; Table S1: details of the sampling locations across the west coast, east coast and central India; Table S2: estimates of the most probable number of genetically distinct groups (K) indicated by STRUCTURE for the *R. rattus* population using the method of Evanno et al., (2005); VCF File: Dataset_*R. rattus*.
