# Supplementary material for: Genomic Analysis Reveals Subdivision of Black Rats (Rattus rattus) in India, Origin of the Worldwide Species Spread
Source: Genes (Basel). 2022 Jan 29;13(2):267. doi: 10.3390/genes13020267 (PMC8871742; doi:10.3390/genes13020267)
Supplement: Supplementary file 1 [file genes-13-00267-s001.zip › Baig et al Electronic supplementary material FINAL.pdf]

Supplementary Material

Genomic analysis reveals subdivision of black rats (*Rattus rattus*) in India, origin of the worldwide species spread

Mumtaz Baig[1, 2, 3,†], Sameera Farah[1,2,†], Ashwin Atkulwar[1,4], Jeremy B. Searle[3*]

1 Laboratory of Molecular and Conservation Genetics, Department of Zoology, Govt. Vidarbha Institute of Science and Humanities, Amravati-444604-India.

2 Department of Integrative Biology, University of Guelph, Guelph, 50 Stone Road East, N1G 2W1, ON, Canada.

3 Department of Ecology and Evolutionary Biology, Cornell University, Corson Hall, Ithaca, NY, 14853-2701, USA.

4 Department of Zoology, Amolakchand Mahavidyalaya, Godhani Road, Yavatmal-445001-India.

† Contributed equally to this work

***Corresponding author: Jeremy B. Searle, telephone: +1-607-254-4236 email: jbs295@cornell.edu**

Figure S1. A STRUCTURE HARVESTER output illustrating the values of delta K for the most probable number of clusters. The highest value of delta K is found for K=2.

Table S1. Details of the sampling locations across the West Coast, East Coast and Central India.

Table S2. Estimates of the most probable number of genetically distinct groups (K) indicated by STRUCTURE for the *Rattus rattus* population using the method of [29]. The significantly favoured number of groups (K=2) is marked by *.

VCF File. Dataset_R. rattus.

Figure S1. A STRUCTURE HARVESTER output illustrating the values of delta K for the most probable number of clusters. The highest value of delta K is found for K=2.

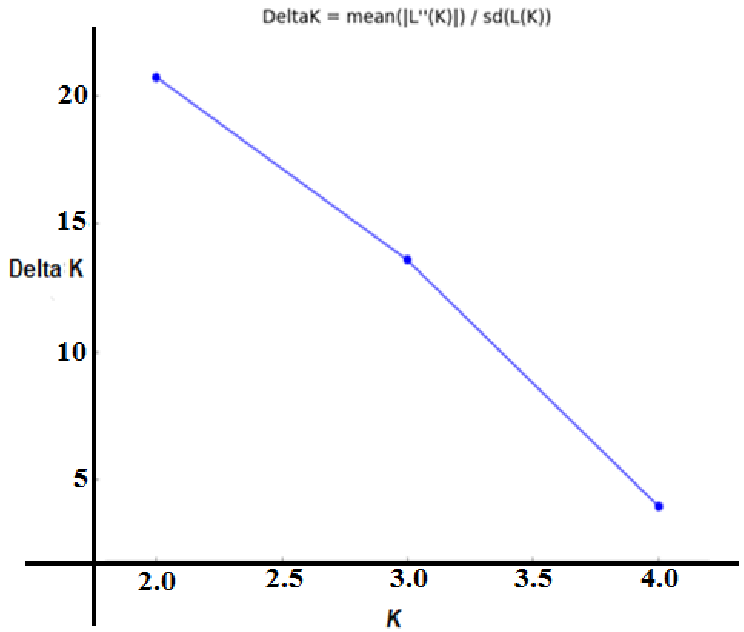

Table S1. Details of the sampling locations across the West Coast, East Coast and Central India.

Specimen	Location	Region (Cluster)
Rr1	Porbandar (Gujarat)	West Coast
Rr2	Porbandar (Gujarat)	West Coast
Rr3	Porbandar (Gujarat)	West Coast
Rr4	Porbandar (Gujarat)	West Coast
Rr5	Navibandar (Gujarat)	West Coast
Rr6	Porbandar (Gujarat)	West Coast
Rr9	Bharuch (Gujarat)	West Coast
Rr12	Surat (Gujarat)	West Coast
Rr13	Surat (Gujarat)	West Coast
Rr14	Surat (Gujarat)	West Coast
Rr18	Thrissur (Kerala)	West Coast
Rr19	Trivandrum (Kerala)	West Coast
Rr27	Brahmapur (Orissa)	East coast
Rr28	Brahmapur (Orissa)	East Coast
Rr29	Vishakhapatnam (Andhra Pradesh)	East Coast
Rr30	Varanasi (Uttar Pradesh)	Gangetic Plain
Rr32	Vishakhapatnam (Andhra Pradesh)	East Coast
Rr33	Vishakhapatnam (Andhra Pradesh)	East Coast
Rr34	Chennai (Tamil Nadu)	East Coast
Rr45	Ratnagiri (Maharashtra)	West Coast
Rr55	Bhopal (Madhya Pradesh)	Central India
Rr56	Bhopal (Madhya Pradesh)	Central India
Rr57	Digha (West Bengal)	East Coast
Rr63	Belgaum (Karnataka)	West Coast
Rr78	Wardha (Maharashtra)	Central India
Rr79	Wardha (Maharashtra)	Central India
Rr80	Wardha (Maharashtra)	Central India

Mm1	(Mouse) Thrissur (Kerala)	West Coast
Mm2	(Mouse) Bharuch (Gujarat)	West Coast
Mm3	(Mouse) Thrissur (Kerala)	West Coast

Table S2. Estimates of the most probable number of genetically distinct groups (K) indicated by STRUCTURE for the *Rattus rattus* population using the method of [29]. The significantly favoured number of groups (K=2) is marked by *.

K	Rep	L (K)	SD	ΔK
1	5	-28738.8600	9.7228	NA
**2**	**5**	**-27286.1800**	**29.9558**	**20.735861***
3	5	-26454.6600	51.5157	13.589266
4	5	-26323.2000	58.4643	3.972680
